# Ethylene, a Signaling Compound Involved in Seed Germination and Dormancy

**DOI:** 10.3390/plants13192674

**Published:** 2024-09-24

**Authors:** Françoise Corbineau

**Affiliations:** Seed Biology, UMR7622 CNRS-Sorbonne-Université, 75005 Paris, France; francoise.corbineau@sorbonne-universite.fr

**Keywords:** ethylene, seed germination and dormancy, crosstalk between ethylene, ABA, Gas and ROS, ethylene signaling pathway, ethylene response factors (ERFs), proteolytic N-degron pathway

## Abstract

The present review is focused on current findings on the involvement of ethylene in seed biology. The responsiveness of seeds to ethylene depends on the species and the dormancy status, improving concentrations ranging from 0.1 to 200 μL L^−1^. The signaling pathway of ethylene starts with its binding to five membrane-anchored receptors, which results in the deactivation of Constitutive Triple Response 1 (CTR1, a protein kinase) that does not exert its inhibitory effect on Ethylene Insensitive 2 (EIN2) by phosphorylating its cytosolic C-terminal domain. An analysis of germination in the presence of inhibitors of ethylene synthesis or action, and using seeds from mutant lines altered in terms of the genes involved in ethylene synthesis (*acs*) and the signaling pathway (*etr1*, *ein2*, *ein4*, *ctr1* and *erf1*), demonstrates the involvement of ethylene in the regulation of seed dormancy. The promoting effect of ethylene is also regulated through crosstalk with abscisic acid (ABA) and gibberellins (GAs), essential hormones involved in seed germination and dormancy, and Reactive Oxygen Species (ROS). Using a mutant of the proteolytic N-degron pathway, *Proteolysis* (*PRT6*), the Ethylene Response Factors (ERFs) from group VII (HRE1, HRE2, RAP 2.2, RAP2.3 and RAP 2.12) have also been identified as being involved in seed insensitivity to ethylene. This review highlights the key roles of EIN2 and EIN3 in the ethylene signaling pathway and in interactions with different hormones and discusses the responsiveness of seeds to ethylene, depending on the species and the dormancy status.

## 1. Introduction

Ethylene (C_2_H_4_) is a simple gaseous molecule with 2C atoms bonded by a double bond; it is considered as a phyto-hormone involved in plant physiology. It is known to be a key regulator of fruit ripening (chlorophyll degradation, climacteric respiratory burst, softening, aroma volatile production, etc.) [1,2,3], but it is also involved in numerous steps of the plant life cycle, including seed germination and seedling development, shoot growth and differentiation, leaf growth and photosynthesis, the induction and opening of flowers, and the senescence and abscission of leaves, flowers, and fruits [4,5,6,7]. It also plays a role in plant tolerance to abiotic stress [5].

Seeds are considered to be dormant when they fail to germinate or germinate poorly when they are placed under apparently favorable conditions (water, temperature, O_2_, light) [8,9,10,11]. The inability to germinate is associated with the embryo itself (embryo dormancy) or can result from the inhibitory action of the covering structures (seed coat-imposed dormancy) [8,9]. Primary dormancy is established during seed development on the mother plant, while secondary dormancy is induced when seeds are placed under unfavorable conditions (too low or too high temperatures, too low oxygen concentration, darkness or light) [9,11]. Baskin and Baskin [12] propose five types of dormancy: (1) physiological dormancy (PD) that can be released by different treatments (chilling, gibberellins, ethylene, etc.); (2) morphological dormancy (MD) due to a delay in embryo development; (3) morpho-physiological dormancy (MPD), combining both PD and MD; (4) physical dormancy (PY) associated with the water impermeability of the seed coat; and (5) combinational dormancy (PY + PD), combining the water impermeability (PY) and embryo dormancy (PD).

Water, oxygen, and temperature are the three crucial factors regulating seed germination. In orthodox seeds, imbibition is a prerequisite to allow the resumption of seed metabolism, including respiration, protein and RNA synthesis, and hormone biosynthesis and signaling pathways [8,13], but other factors such as temperature and oxygen are essential. Seed sensitivity to environmental factors (oxygen, temperature) depends on the species and the dormancy depth [8,14]. In addition, the requirement of oxygen depends on the nature of the reserve (lipids, starch, proteins) and the temperature [14,15], and the temperature range allowing germination is associated with the seed origin. The optimum temperatures for germination are around 10–20 °C and 25–35 °C for seeds from temperate climates and tropical and sub-tropical climates, respectively [8,16]. Generally, non-dormant seeds germinate in a wider range of temperature than dormant ones [8,16,17].

The regulation of seed dormancy and germination by the hormonal balance between abscisic acid (ABA) and gibberellins (GAs) is well documented [18,19,20,21,22,23,24,25]. ABA is known to play a crucial role in the induction of dormancy during seed development and its maintenance during seed imbibition, while GAs are involved in dormancy release or germination [19,20,26,27]. In addition to ABA and GAs, other hormones (ethylene, brassinosteroids, jasmonic acid, auxins, cytokinins, etc.) are involved in the control of seed germination and dormancy [28,29,30,31,32] via a complex hormonal signaling network.

From the past decade onwards, Reactive Oxygen Species (ROS) have also been recognized as key regulators of cellular signaling pathways and have emerged as fundamental actors in seed germination and dormancy [33,34,35,36]. The interaction of ROS with the ABA, GA, and C_2_H_4_ signaling pathways has been demonstrated in the regulation of dormancy in sunflower [37,38,39,40], barley [41] and Arabidopsis [42,43].

The aims of the present review are (1) to describe the responsiveness of dormant seeds to ethylene and the involvement of its biosynthesis and signaling pathway in dormancy release using different mutants; (2) to discuss the interactions between C_2_H_4_ and other hormones, particularly ABA and GAs, and ROS; and (3) to demonstrate the emerging mechanisms that contribute to the regulation of seed dormancy by C_2_H_4_ by highlighting the involvement of the proteolytic N-degron pathway in response to C_2_H_4_, with group VII of the Ethylene Response Factors being substrates of this pathway.

## 2. Effects of Exogenous Ethylene on Seed Germination

The effects of ethylene on seed germination and dormancy are well documented [14,30,31,32,44,45]. Ethylene stimulates the germination of seeds from various species, including several parasitic plants such as *Orobanche ramosa* [46] and some striga (*Striga asiatica*, *S. lutea*, *S. hermonthica*) [47,48], numerous weeds, including the model plant *Arabidopsis thaliana*, and cultivated plants from the Rosaceae (apple, *Malus domestica*; peach, *Prunus persica*; bird cherry, *Prunus avium*), Asteraceae (sunflower, *Helianthus annuus*; lettuce, *lactuca sativa*), Amaranthaceae (redroot-pigweed, *Amaranthus retroflexus*; beet, *Beta vulgaris*), and Fabaceae families (peanut, *Arachis hypogaea*) (Table 1, [14,30,31,32,44,45]). This hormone allows germination of seeds exhibiting embryo dormancy to occur, like apple [49,50,51,52,53], peach [54], bird cherry [55], beechnut [56], and sunflower [57,58]. It replaces chilling, which usually breaks dormancy in Rosaceae seeds and beechnut, and after-ripening in dry conditions, which alleviates dormancy in sunflowers. In the case of sunflower, dormant embryos become able to germinate at low temperatures (10–15 °C) in the presence of ethylene (20–50 μL L^−1^) when they cannot germinate in air [57]. Ethylene also removes seed coat-imposed dormancy in various species (*Arabidopsis thaliana* [59,60,61,62], *Rumex crispus* [63,64], *Trifolium subterraneum* [65], *Xanthium pennsylvanicum* [66,67,68,69]) (Table 2, [14,30,31,32,44,45,49,50,51,52,53,54,55,56,57,58,59,60,61,62,63,64,65,66,67,68,69,70,71,72,73,74,75,76,77,78,79,80,81,82,83,84,85,86,87,88]). In addition, the inhibitory effect of high temperatures (thermo-dormancy) is alleviated by C_2_H_4_ in seeds of lettuce [82], *Cicer arietinum* [79], and sunflower [80]. Ethylene also promotes germination at non-optimal temperatures, i.e., at high temperatures, in *Xanthium pennsylvanicum* [68], lettuce [83,84], and *Amaranthus retroflexus* [74], suggesting that it prevents the induction of secondary dormancy induced by high temperatures.

The stimulatory effect of ethylene is dose dependent, and optimal concentrations range from 0.5 to 200 μL L^−1^, depending on the species and the dormancy depth. The sensitivity to ethylene increases during the breaking of seed dormancy either during chilling or dry storage (after-ripening). In the case of sunflower, for example, the dormant embryos at harvest require 40–50 μL L^−1^ ethylene in order to germinate at 15 °C, and only 3 μL L^−1^ after 15 weeks of dry storage [89]. In Arabidopsis (Col 0) seeds, which exhibit a seed coat-imposed dormancy at temperatures higher than 10 °C, 100% germination occurs at 25 °C in darkness in the presence of 50–100 μL L^−1^ ethylene, but after 1 day at 4 °C, 1.25 μL L^−1^ ethylene is enough to allow 75% germination [62]. Ribeiros and Barros [88] demonstrated that non-dormant seeds of *Stylosanthes humilis* are at least 50-fold more sensitive to ethylene than freshly harvested dormant ones. In contrast, sensitivity to ethylene decreases during the induction of a secondary dormancy [68,69,81,89,90].

The effect of ethylene also depends on the time at which seeds are imbibed in its presence. Ethylene is most effective during the first 4 h of imbibition in *Xanthium pennsylvanicum* [66], when the effectiveness of a 3 h treatment in the presence of 50 μL L^−1^ ethylene increases during sunflower seed imbibition; this effect is optimal after about 2 days, and then decreases [89]. In addition, the improving effect of ethylene also requires at least 5% oxygen in sunflower [58] and Arabidopsis seeds [62,91].

Often, ethylene stimulates the germination of seeds which are light sensitive; however, the hormone generally enhances the effect of light, but does not overcome the light requirement [14].

Different studies have also indicated that ACC, the direct precursor of ethylene, applied in the medium at a concentration of 0.1–1 mM, stimulates the germination of dormant seeds of *Arabidopsis thaliana*, *Helianthus annuus*, and *Lactuca sativa* (cf. references in Table 2).

## 3. Ethylene Biosynthesis and Seed Germination

### 3.1. Ethylene Biosynthesis Pathway

**Figure 1:**
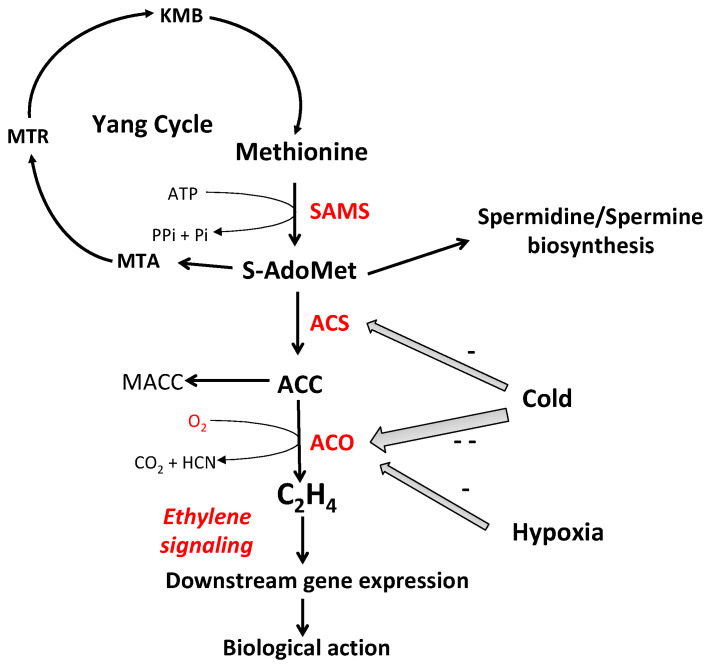
Ethylene (C_2_H_4_) biosynthesis pathway. ACC, 1-Aminocyclopropane-1-carboxylic acid; ACO, ACC oxidase; ACS, ACC synthase; KMB, 2-keto-4-methylthiobutyrate; MACC, Malonyl-ACC; MTA, 5-Methylthioadenosine; MTR, 5-Methylthioribose; S-AdoMet, S-adenosylmethionine. Cold differentially inhibits ACS and ACO activities, resulting in ACC accumulation during cold treatment and in an ethylene burst after transfer of the organs at warmer temperatures. Hypoxia inhibits ACO activity, which is nil in anoxia, with oxygen being required for ACO activity. From [28,30,31,32,92].

Ethylene biosynthesis in seeds is the same as that described for other organs (leaves, flowers, fruits, etc.) [4,28,92,93]. It consists in three steps (Figure 1): in the first step, the amino acid, methionine, is converted to S-Adenosyl-L-Methionine (S-AdoMet or SAM) via the methionine adenosyltransferase or SAM synthetase (SAMS); In the second step, S-AdoMet is converted to 1-Aminocyclopropane-1-Carboxylic acid (ACC), the precursor of ethylene, and 5′-Methylthioadenosine (MTA) by ACC-synthase (ACS). This biochemical reaction is considered to be the rate-limiting step during ethylene biosynthesis [92]. MTA is then recycled back to methionine through the Yang cycle [93]; In the last step, ACC oxidation by ACC oxidase (ACO) results in the production of C_2_H_4_ with carbon dioxide (CO_2_) and hydrogen cyanide (HCN) as by-products [94,95]. This reaction requires oxygen as a co-substrate [93,96,97]. The apparent Km for ACO activity measured in vivo and in vitro ranges from 0.4 to 10–11% oxygen, depending on the plant organ, ACC concentration, and temperature [96,97]. In sunflower hypocotyl segments, the Km value for oxygen is similar for ACO measured in vitro (11.4%) and in vivo (10.6%) [98]; however, it is much higher than the Km value measured in melon [99] and avocado fruit [100]. ACC can also be converted to 1-(Malonylamino)-Cyclopropane-1-Carboxylic acid (MACC) [101]. Polyamine biosynthesis can occur after decarboxylation of S-AdoMet via the S-Adenosyl-Methionine decarboxylase.

ACS and ACO belong to a multi-gene family, and the expression of *ACS* and *ACO* genes differ among each other [95,102,103]. In Arabidopsis, there are height functioning *ACS* genes [103,104]. For ACO, homology analyses revealed five *AtACO* genes, but ACO1 and ACO2 seem to be the major ACOs in seeds in two Brassicaceae (*Arabidopsis*, *Lepidium sativum)* [105,106]. Ethylene has been shown to regulate its own synthesis via a biochemical process known as autocatalytic biosynthesis, by inducting *ACO* transcription [107]. It is also required to stimulate *ACO* gene expression in pea [108,109], beechnut [56], and turnip [110]. In contrast, the expression of *SoACS7* in *Sisymbrium officinale* and *PsACS1* in pea is not affected [86,108,109]. Among the treatments which break dormancy, cyanide, which alleviates apple and sunflower embryo dormancy, also stimulates the ethylene production associated with an increase in ACS and ACO activities in apple seedlings [111], while it reduces *HaACS* and *HaACO* expression in sunflower [112]. In Arabidopsis, *ACS6* is activated in response to cyanide when other *ACS* gene are unaffected [113]; in such cases, chilling down-regulates the expression of *ACOs* but results in the transient expression of *ACS* [29,31,114]. Cold stratification is often associated with an improvement of ethylene production after transfer to a warmer temperature due to an accumulation of ACC resulting from a differential inhibition of cold on ACS and ACO (Figure 1).

### 3.2. Ethylene Production during Germination

The increase in ethylene production in germinating seeds is essentially explained by an increase in ACC oxidase activity [28,29,31,32,44,86]. Ethylene production is generally low during phases 1 (imbibition) and 2 (germination *stricto sensu*) of the germination process, and a burst of the gas is detected during phase 3 (growth), when the radicle elongates through the seed coat [40,75,115,116]. In sunflower embryos, for example, using a laser photo acoustic spectroscopy, El-Maarouf-Bouteau et al. [40] measured ethylene emanation of about 0.1 mL h^−1^ g dw^−1^ in both dormant and non-dormant embryos.

The stimulatory effects of exogenous ethylene in the breaking of seed dormancy in numerous species (cf. Table 1 and Table 2) suggest that this compound is involved in seed physiology. This involvement was also shown and confirmed by using inhibitors of ethylene biosynthesis affecting ACC activity (AVG, Amino-ethoxyvinylglycine; AOA, Amino-oxyacetic acid) and ACO activity (Co^2+^). AVG inhibits ethylene production, but its effect on germination depends on the species. It also inhibits the germination of apple embryos [50] but does not have a strong action on the germination of lamb’s quarters seeds [78], bean [117], peanut [118], or *Amaranthus caudatus* [70]. The inhibitory effect of AVG disappears in the presence of exogenous ethylene.

The effects of a few antagonists of the action of ethylene (CO_2_; 2,5-NBD, 2,5-Norbornadiene; STS, Silver Thiosulfate) have been studied in numerous species. However, the effect of CO_2_ is debatable, since it can inhibit and/or stimulate seed germination [44]. The inhibitory effect of STS has been shown in sunflower [57,58] and thermo-dormant lettuce [82]. More recently, the use of 2,5-NBD, a volatile cyclic olefin that inhibits the action of ethylene at its binding site, revealed that endogenous ethylene was required to break dormancy or improve seed germination [70,71].

Ethylene production remains low during seed imbibition and generally becomes detectable when radicles protrude from the seed covering structures at the end of the germination process [40,119,120]. Radicle protrusion is always associated with a peak of ethylene emanation. Similarly, *ACO1* expression in pea [109] and in turnip [121] is maximal at radicle emergence. A close relationship between ethylene production and seed vigor has also been reported in numerous species such as snapbean [122], pea, cocklebur [123], and sunflower [124]. ACC-dependent C_2_H_4_ production has therefore been proposed as a marker of seed quality [125,126].

Exogenous ACC stimulates the germination of various ethylene-sensitive seeds such as lettuce, sunflower, cocklebur, *Amaranthus caudatus* and *A. retroflexus*, chickpea, and sugar beet (Table 2, review by [31]). This stimulatory effect of ACC suggests that dormancy is related to low ethylene production due to an insufficient level of ACC. Table 3 ([57,58]) shows the respective effects of ethylene, ACC, and various inhibitors of ethylene synthesis, as well as their actions on the germination of dormant and non-dormant embryos of sunflower. Both ACC and exogenous ethylene were shown to stimulate the germination of dormant embryos, when the various inhibitors used markedly inhibited the germination of non-dormant ones. All these results suggest that ethylene is involved in the germination of dormant and non-dormant seeds.

## 4. Ethylene Signaling Pathways

### 4.1. Canonical Ethylene Signaling Pathway

**Figure 2:**
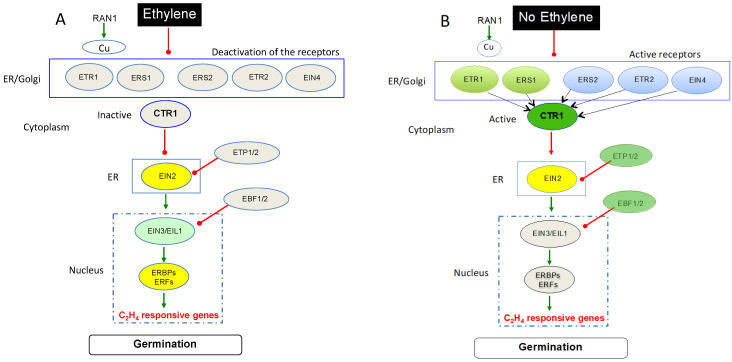
Schematic model of the ethylene signaling pathway in the presence (**A**) and in the absence of ethylene (**B**). The ethylene receptors localized at the endoplasmic reticulum (ER) are divided into two subfamilies based on the feature of the histidine kinase motif; subfamily I includes ETR1 and ERS1, while ETR2, ERS2 and EIN4 fall into subfamily II. CTR1 interacts with the five receptors, but it is presented separately in order to indicate its state (active or inactive). In the presence of C_2_H_4_ (**A**), the receptors deactivate CTR1, which, in turn, activates EIN2 that interacts with EIN3 and EIL1, allowing it to activate ethylene response factors (ERFs), resulting in the expression of ethylene responsive genes. EBF 1 and 2 (EIN3-binding F box protein 1 and 2) are able to inhibit EIN3 and EIL1. Without ethylene (**B**), the receptors-CTR1 complexes maintain CTR1 in an active state, allowing it to phosphorylate EIN2, thus preventing ethylene response through EIN2. In the nucleus, the EIN3/EIL1 are degraded. The turnover of EIN2 is regulated by ETP and the degradation of EIN3 is controlled by EBF. CTR1, Constitutive Triple Response 1; EBF, EIN3 Binding F-Box; EIL3, Ethylene Insensitive-Like protein; EIN2, Ethylene Insensitive 2; EIN3, Ethylene Insensitive 3; EIN4, Ethylene Insensitive 4; ERBPS, Ethylene Responses Element Binding Proteins; ERFs, Ethylene Response Factor; ERS1, ERS2, Ethylene Response Sensor 1 and 2; ETP, EIN2 Targeting protein; ETR1, ETR2, Ethylene Resistant 1 and 2; RAN, Response to Antagonist 1, a copper cofactor. An activated component is shown by color, while an inactivated or repressed component is shown in grey. Modified from [128,130,131,132,133].

The ethylene signaling pathway includes several key components, including a family of five membrane receptors of the hormone, CTR1 (CONSTITUTIVE TRIPLE RESPONSE 1), a negative regulator of the pathway, EIN2 (ETHYLENE INSENSITIVE 2), a central positive regulator of the pathway, and EIN3 (ETHYLENE INSENSITIVE 3) and its homolog EIN3-Like (EIL1) that accumulate in the nucleus and initiate a transcriptional cascade involving Ethylene Responses Factors (ERFs) controlling the expression of ethylene dependent genes (Figure 2, [7,127,128,129,130,131,132,133]). In Arabidopsis, C_2_H_4_ is recognized by five receptors: ETR1 and ETR2 (ETHYLENE RESPONSE 1 and 2), ERS1 and ERS2 (ETHYLENE RESPONSE SENSOR 1 and 2), and EIN4 (ETHYLENE INSENSITIVE 4), localized in the membrane of the endoplasmatic reticulum. These receptors are divided into two groups. The first group consists of ETR1 and ERS1, which are characterized by a histidine kinase in their N terminal end and by three trans-membrane domains that bind C_2_H_4_ through coordination with copper [134,135]; the second group includes ETR2, ERS2, and EIN4, which possess four trans-membrane regions but contain degenerate histidine kinase domains and exhibit serine/threonine kinase activity [129,132,136,137,138]. Genetic study using different mutants, i.e., single (*etr1*), double (*etr1*, *ers1*) and triple (*etr2*, *ers2*, *ein4*), demonstrated that all receptors are able to sense ethylene, but ETR1 and ERS1 are suggested to be more important than the three other receptors [133,139,140]. Some convergences have also been revealed between AHK2-4 (ARABIDOPSIS HISTIDINE KINASE 2-4), which functions as a cytokinin receptor, and ETR1 and ERS1, two ethylene receptors [18,141].

In the presence of ethylene (Figure 2A), the binding of C_2_H_4_ to receptors is facilitated by copper supplied by a copper transporter (RAN1, RESPONSIVE TO ANTAGONIST 1), resulting in the deactivation of the histidine kinase activity of CTR1 which, in turn, leads to the activation of the signaling compound EIN2, considered to be a central component in the hormone signal transduction pathway [127,142,143]. The loss of sensitivity to ethylene of *ein2* mutant confirms this key role [127,131,144]. The cleavage of the C-terminal end of EIN2 and its translocation to the nucleus activates downstream components of the pathway [144]. Consequently the primary transcription factors (EIN3 and EIN3-like = EIL) are activated, which, in turn, activate other transcription factors, called Ethylene Response Factors (ERFs). The turnover of EIN2 is regulated by ETP1 and ETP2 (EIN2 TARGETING PROTEIN) [145], and the EIN3 level is controlled by EBF (EIN3 BINDING F-BOX) [146,147,148]. The ERFs bind to the promoter region of ethylene-responsive genes, resulting in an altered gene regulation, and in the nucleus, EIN3, EILs, Ethylene Response Element Binding Proteins (EREBPS) and ERFs activate the transcription of ethylene-response genes [129,132,133,149].

In the absence of ethylene (Figure 2B), receptor-CTR1 complexes remain active, allowing CTR1 to phosphorylate EIN2, thus preventing ethylene response through EIN2. The stability of EIN2 is regulated by ETP1/2 through interaction with its C-terminal. In addition, in the nucleus, EIN3 also can be degraded by EBF1/2 [130,132,145].

Although the molecular mechanisms of the transduction of ethylene signaling have been studied extensively, works carried out in the H. Qiao laboratory (University of Texas, Austin, TX, USA) have provided evidence that chromatin modifications, specifically histone acetylation, regulate ethylene signaling and response [150,151,152,153,154,155]. In the presence of ethylene, the EIN2 C-terminus contributes to downstream ethylene signaling via elevation of acetylation at H3K14 and H3K23, depending on both EIN2 and EIN3 [150,151]. The authors propose a unique mechanism by which ENAP1 (EIN2 nuclear associated protein 1) interacts with chromatin: in the absence of ethylene, ENAP1 interacts with histones, preserving the open chromatin regions, while in the presence of ethylene, it interacts with EIN2 C-terminus, resulting in elevated acetylation levels and leading to fast transcriptional responses [152,153,154,155]. In addition, the identification of two histone deacetylases (HDACs), SRT1 and SRT2, that interact with ENAP1 also allows us to conclude that acetylation and deacetylation switch on and off gene expression in response to ethylene [153,154,155].

### 4.2. Non Canonical Ethylene Signaling Pathways

**Figure 3:**
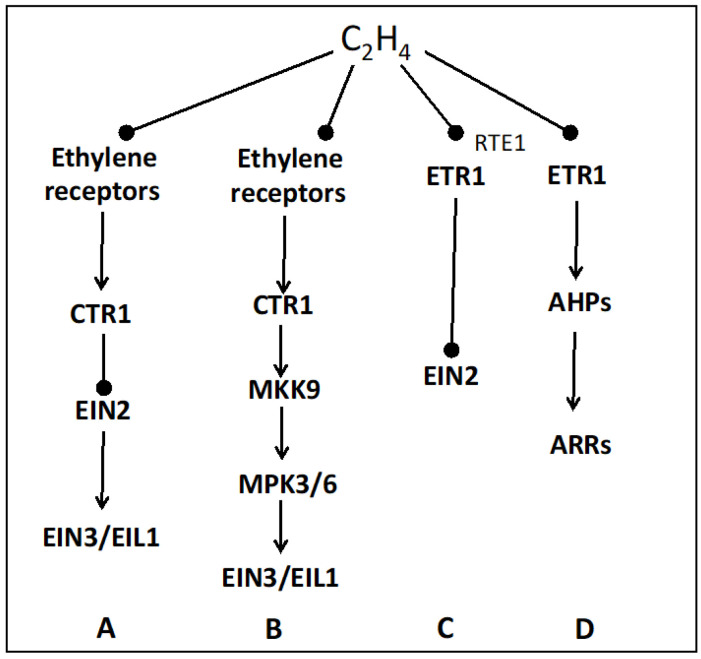
Ethylene signaling transduction pathways. A, the canonical ethylene signaling (cf. Figure 2); B, EIN2 independent signaling pathway involving a MAPK cascade; C, CTR1 independent signaling pathway involving RTE1; D, two-component signaling that relies on the histidine kinase activity of the receptor. AHPs, Arabidopsis Histidine-containing Phosphotransmitter; ARRs, Arabidopsis Response Regulators; CTR1, Constitutive Triple Response; EIN2, Ethylene Insensitive 2; EIN3, Ethylene Insensitive 3; EILs, EIN Like; ETR1, Ethylene Response 1; MKK9, Mitogen-Activated Protein Kinase Kinase; MPK3/6 Protein Kinase 3/6; RTE1, Reversion To Ethylene sensitivity 1. Modified from [91,130,159,165].

In the past, studies on the ethylene signaling pathway have suggested the existence of signaling pathways where ethylene receptors could initiate an alternative signaling pathway independently of CTR1 or EIN2 [133,156,157]. Figure 3 summarizes the structure of the classical ethylene signaling pathway (Figure 3, line A) and indicates three alternative pathways (Figure 3, lines B, C and D). The EIN2 independent pathway (Figure 3, line B) involves a mitogen-activated protein kinase (MAPK) cascade that bypasses EIN2 and acts downstream of CTR1. MKK9 is reported to activate MAP3/6, which then phosphorylates EIN3 [130]; however, the role of the MAPK cascade remains unclear [133,158]. In the signaling pathway described in Figure 3 (line C), RTE1 (REVERSION TO ETHYLENE SENSITIVITY 1) is required for ETR1 N-terminal signaling [159,160,161], and EIN2 is suggested to be the potential candidate [162,163]. The last alternative pathway (Figure 3, line D), named Two-Component signaling (TCS), involves histidine kinases and a response regulator. The mediated proteins are referred to as Arabidopsis Histidine-containing Phosphotransmitters (AHPs) that target at the Arabidopsis Response Regulators (ARRs) [164], subsequently affecting ethylene signaling [165]. This TCS pathways seems to be involved in the regulation of hypocotyl growth, stomatal closure regulation, shortening root, and plant defense [166,167,168,169].

### 4.3. ERFs

Downstream signaling of ethylene relies on the activation of a large array of ERFs which, in turn, modify gene expression [43]. ERF1 that acts downstream of all the components of the ethylene signaling (cf. Figure 2) was the first gene identified in this family that includes 147 members in Arabidopsis and is subdivided into twelve groups [170]. It acts as the intermediate between EIN3 and ethylene-inducible target genes. Several proteins in the ERF family are targets of EIN3 and are also induced by C_2_H_4_, such as ERF2, ERF5, and ERF11 [171]. They have also been shown to regulate the expression of the genes involved in dehydration and hypoxia responses [172,173] and in seed dormancy regulated through DOG1 (DELAY OF GERMINATION) [174].

It is important to note that the ERFs from group VII consist of five proteins, i.e., RAP 2.2 (RELATED TO AP), RAP 2.3, RAP 2.12, HRE1 (HYPOXIA RESPONSE), and HRE2, nominated ERF 75, 72, 74, 73 and 71, respectively [170], and identified as substrates of the N-degron pathway [175]. Recently, Wang et al. [62,176] demonstrated that mutant seeds affected in the proteolytic N-degron pathway, proteolysis (*prt6*), are insensitive to ethylene, suggesting that PRT6 is involved in the dormancy release by ethylene in Arabidopsis. ERFs from group VII have also been found to be involved in seed insensitivity to ethylene. Furthermore, exogenous ethylene reduces the expression of the three RAPs in wild type (Col-0) seeds but induces or maintains their expression in *prt6* mutant.

## 5. Crosstalk between Ethylene, Plant Hormones, and ROS

### 5.1. Interrelation between Ethylene, ABA, and GA

Ethylene plays an important role in seed dormancy alleviation and germination through the regulation of the ABA/GA hormonal balance as well as the control of the expression of various genes involved in ABA, GAs, and other hormone (jasmonic acid, auxins, cytokinins) biosynthesis and signaling pathways [31,62,105,177]. There is a negative interaction between ABA and C_2_H_4_, and the inhibitory effect of ABA on the germination can be partially reversed in the presence of C_2_H_4_ or ACC, the precursor of ethylene [105,178,179,180]. Treatment for 30 h with 100 μL L^−1^ ethylene of dormant Arabidopsis seeds imbibed at 25 °C resulted in the germination of all of the seed population associated with a decrease in ABA content from 43.9 ng g DW in air to 6.2 ng g DW in the presence of ethylene [176]. ACC oxidase (ACO) is up-regulated in *aba2* mutant, whereas both the ABSCISIC ACID INSENSITIVE (ABI1) and cytochrome P450, family 707, (CYP707A2) genes are down-regulated in *etr1-1* [181]. The improving effect of ethylene is also associated with a decrease in seed sensitivity to ABA [178]. Moreover, mutations in the ethylene synthesis pathway often increase sensitivity to ABA [182] and, in parallel, mutations in both synthesis and signaling pathways affect seed sensitivity to C_2_H_4_ [29,30,177,178,181]. For example, the emission of ethylene is one third that of wild type in *acs 7* mutant. The seeds also become hypersensitive to ABA [182].

Regarding the interactions between C_2_H_4_ and gibberellins, GAs are able to stimulate the germination of seeds, the dormancy of which is broken by ethylene, and there is a positive interaction between both hormones [28,31,44,60,177,183]. Ethylene mimics the action of GAs, since it improves the germination of GA-deficient mutants of tomato (*gib1*) and Arabidopsis (*ga1*) [184]. In Arabidopsis, the improving effect of ethylene has been associated with a decrease in ABA (see above) and an increase in GA_4_ from 0.49 to 1.08 ng g DW after 30 h of imbibition in the presence of ethylene, resulting in a decrease of ABA/GA_4_ ratio from 89.7 in air down to 5.7 in the presence of ethylene [176]. This beneficial effect is associated with a down-regulation of the expression of ABI 5 (ABA INSENSITIVE 5), a core component of the ABA signaling pathway, and of RGL2 (RGA-Like 2) and RGA 2 (REPRESSOR OF *ga1-3*) negative regulators of GA signaling [176].

### 5.2. Interrelation between Ethylene, ABA and GA Signaling Pathways

Studies using mutant lines altered in the genes involved in the ethylene signaling pathway (*etr1*, *ein2*, *ain1,* and *erf1*) demonstrated the involvement of C_2_H_4_ in the regulation of seed dormancy through a complex hormonal signaling network [18,28,31,185]. Mutation at the level of C_2_H_4_ receptor (ETR1) *etr-1* leads to a deeper dormancy than the wild type and impacts sensitivity to ABA and ABA content [59,60,61,178,179,181,186,187,188,189] (Table 4). It also results in higher endogenous ABA content than in wild type, and in a lower level of ABA glucose ester (ABA-GE), suggesting that reduced ABA metabolism contributes to ABA accumulation in the mutant [187]. This increase in ABA content could also result from a down-regulation of CYP707A2 in *etr1-1* [181]. The mutation of *etr1-2* also results in an increase in GAs, in auxin (IAA) and its metabolite indole-3-aspartate (IAAsp), and in cytokinin [187].

EIN2 plays a key role in the C_2_H_4_ signaling pathway, and similarly to seeds of *etr1*, seeds of *ein2* mutant are insensitive to ethylene, exhibit a deeper dormancy than the wild type, and are ABA hypersensitive [178,179,181,187,188,189] (Table 4). The high ABA level in *ein2* mutant is due to the up-regulation of NCED3, a key enzyme in the ABA biosynthesis pathway [181]. It has been proposed that EIN2 lies at the cross-roads of multiple hormone response pathways [127]. Conversely, mutation in *ctr1* leads to an increased ethylene response and a decrease in ABA sensitivity [189] (Table 4). In addition, seeds of *ctr1* mutant have a slightly enhanced rate of germination [28] (Table 4).

The stimulatory effect of C_2_H_4_ is associated with an activation of GA synthesis and signaling, while the promoting effect of GAs is often due to a stimulation of C_2_H_4_ synthesis [29,86]. For example, in the case of *ga1-3* mutant (GA-deficient mutant), GA_4_, which stimulates seed germination, also up-regulates the expression of ACO and ERS1 (ETHYLENE SENSOR 1) [183]. Studies of the expression of *SoGA3ox2* and *SoGA20ox2*, two genes involved in GA biosynthesis, during imbibition of *Sisymbrium officinale* in the presence of GA_4+7_ and ethylene, indicated that GA synthesis is strongly regulated by both GA and ethylene [86]. In contrast, in beechnut embryos, the expression of *FsGA20ox1* is increased when ethylene synthesis is inhibited in the presence of 2-aminoxyacetic acid (AOA) [56].

**Figure 4:**
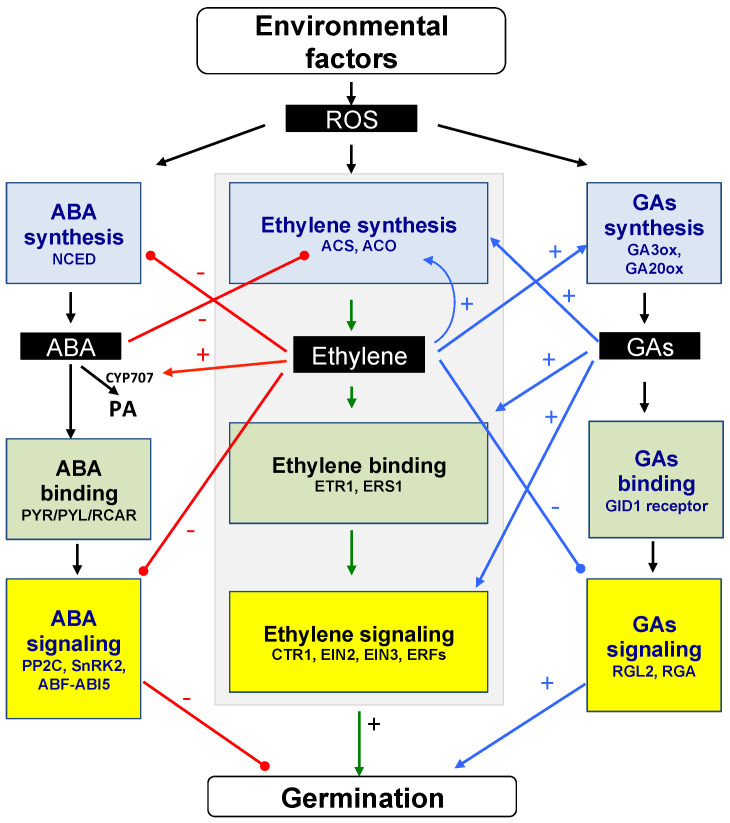
Interactions between ethylene, abscisic acid, gibberellins, and ROS in the regulation of seed germination. This scheme is based on genetic, molecular, and physiological studies cited in the text on seed responsiveness to C_2_H_4_, ABA, GAs, and ROS. Ethylene down-regulates ABA level by inhibiting its synthesis, in particular through NCED, and promoting its catabolism through CYP707. It also down-regulates ABA signaling through ABI5. In addition, ABA inhibits ethylene biosynthesis by reducing ACS and ACO activities. Ethylene also improves GA metabolism, through GA3ox and GA20ox, and GAS signaling, through RGL2 and RGA. ROS produced in relation with stress environments enhance ABA catabolism and both C_2_H_4_ and GAs signaling pathways (black arrows). (+) and (−) indicate positive and negative interactions between the components of the signaling cascade. The interrelations between ABA and ethylene, and GAs and ethylene, are in red and blue, respectively. ABA, abscisic acid; ABF, ABA-response element-(ABRE) binding factors; ABI5, ABA INSENSITIVE 5; ACC, 1-aminocyclopropane-1-carboxylic acid; ACO, ACC oxidase; ACS, ACC synthase; CTR1, Constitutive Triple Response 1; CYP707, cytochrome P450-dependent mono-oxygenase; EIN2, Ethylene Insensive 2; EIN3, Ethylene Insensitive 3; ERFs, Ethylene Response Factors; ERS1, Ethylene Response Sensor; ETR1, Ethylene Response 1; GAs, gibberellins; GA3ox, GA3-oxidase; GA20ox, GA20-oxidase; GID1, receptor GIBBERELLIN INSENSITIVE DWARF1; NCED, 9-cis-epoxycarotenoid dioxygenase; PA, phaseic acid; PP2C, phosphatase 2C proteins; PYL, PYR1-like; PYR, pyrabactin resistance; RCAR, regulatory components ABA receptor; RGA, REPRESSON OF *ga-1-3*; RGL2, RGA LIKE 2; SnRK2, subfamily 2 SNF1-related kinase. Modified from [31].

Figure 4 illustrates the interactions between ethylene, ABA, and GAs based on genetic, molecular, and physiological studies on seed responsiveness to the three hormones and cited in Section 5.1 and Section 5.2. Ethylene down-regulates the ABA content by inhibiting its synthesis, in particular through NCED and the promotion of its catabolism through CYP707. It also regulates ABA signaling through ABI5. In addition, ABA inhibits C_2_H_4_ biosynthesis by reducing ACS and ACO activities. Ethylene also improves GA metabolism, through the regulation of GA3ox and GA20ox activities, and GA signaling pathways, through RGL2 and RGA.

### 5.3. Interrelation between Ethylene and ROS

Reactive oxygen species (ROS) and reactive nitrogen species, such as hydroxyl radical (·OH), hydrogen peroxide (H_2_O_2_), superoxide anion (O_2_^−^), hydroxylamines, and nitrates, break the dormancy of different species [34,37,38,76,190,191]. ROS are considered as key signaling compounds in the regulation of seed dormancy and germination ([33,34,35,36], and interrelations with ABA, GAs, and C_2_H_4_ have been established in sunflower [40], barley [41], and Arabidopsis [42,43]. In sunflower, C_2_H_4_ breaks dormancy [57], and this improving effect is associated with an increase in ROS accumulation within the embryonic axis, probably through the activation of NADPH oxidase [40]. In contrast, Lin et al. [192,193] showed that ethylene treatment of Arabidopsis seeds imbibed under salinity stress resulted in a decrease in ROS level. These conflicting results may be explained in part by the high production of ROS in response to stress [33,35]. Numerous works have investigated the effects of ROS on C_2_H_4_ production during germination; however, the data obtained are often questionable, since C_2_H_4_ emission peaks during radicle protrusion through the seed envelops, i.e., after the germination process itself [40]. The synergistic effects of ROS and C_2_H_4_ on the breaking of dormancy has been demonstrated in different species, such as *Sorbus pohuashanensis* [194], wild cardoon [195], and *Brassica oleracea* [76].

Recently, Jurdak et al. [43] found that seed response to ethylene involved mitochondrial retrograde response through nuclear ROS production and the up-regulation of the AOX1a (alternative oxidase) and ANACO13 (transcription factor of the NAC (NAM, ATAF, and CVC) families.

ROS production regulated by environmental conditions enhances ABA catabolism and both ethylene and GAs signaling pathways (Figure 4).

## 6. Conclusions and Future Research Directions

Seed germination and dormancy are multifactorial phenomena, the regulation of which involves different internal and external factors. Among these factors, the soil atmosphere, and particularly ethylene, may exert important effects on seed physiology. The interactions between ethylene, ABA, and GAs have been quite well described, but it would also be important to determine the hierarchy of the components of the signaling pathways of various other hormones, such as brassinosteroids, jasmonate, and auxins, and to identify their putative role as sensors of environmental signals. Analyses of data obtained using proteomic, metabolomic, and transcriptomic tests must also take into account the complexity of the crosstalk between ethylene and other hormones and ROS. Bioinformatic approaches would then also be important to integrate the complexities of the different networks. Recently, the N-degron pathway was demonstrated to be involved in the response of Arabidopsis seeds to exogenous ethylene. It is also necessary to identify the direct effect of ethylene and its indirect effects in relation with the metabolites produced in parallel, such as polyamides after decarboxylation of S-AdoMet, and CO_2_ and CN, byproducts of ACC oxidation, and the effects on other hormone syntheses and signaling pathways.

## Figures and Tables

**Table 1:** Some species in which germination is stimulated or seed dormancy is broken by exogenous ethylene or ethephon, an ethylene-releasing compound. Modified from [14,30,31,32,44,45].

Parasitic Plants	Weeds	Cultivated Plants
*Orobanche ramosa* *Stiga asiatica* *Striga hermonthica* *Stiga lutea*	*Amaranthus caudatus* *Arabidopsis thaliana* *Chenopodium album* *Rumex crispus* *Xanthium pennsylvanicum* *Spergula arvensis*	*Amaranthus retroflexus* *Arachis hypogea* *Beta vulgaris* *Brassica oleracea* *Helianthus annuus* *Lactuca sativa* *Malus domestica* *Prunus avium* *Prunus persica*

**Table 2:** Plant species in which the primary or secondary seed dormancies is broken by exogenous ethylene or ethephon, an ethylene-releasing compound. Modified from [14,30,31,32,44,45].

Type of Dormancy	Species		References
Primary and secondary dormancies	*Amaranthus caudatus*	Love-lies-bleeding	[70,71]
Secondary dormancy	*Amaranthus paniculatus*	Red amaranth	[72]
Primary dormancy	*Amaranthus retroflexus*	Redroot-pigweed	[67,73,74]
Primary dormancy	*Arabidopsis thaliana*	Arabidopsis	[59,60,61,62]
Primary dormancy	*Arachis hypogaea*	Peanut	[75]
Primary dormancy	*Brassica oleracea*	Chinese cabbage	[76]
Primary dormancy	*Chenopodium album*	Lambs’ quarters	[77,78]
Thermo-dormancy	*Cicer arietinum*	Chick-pea	[79]
Primary dormancy	*Fagus sylvatica*	Beechnut	[56]
Primary and secondary dormancies	*Helianthus annuus*	Sunflower	[57,58,80]
Thermo- and secondary dormancies	*Lactuca sativa*	Lettuce	[81,82,83,84]
Primary dormancy	*Prunus avium*	Bird cherry	[55]
Primary dormancy	*Prunus persica*	Peach	[54]
Primary dormancy	*Pyrus malus*	Apple	[49,50,51,52,53]
Primary dormancy	*Rhus coriaria*	Sicilian sumac	[85]
Primary and secondary dormancies	*Rumex crispus*	Curly dock	[63,64]
Primary dormancy	*Sisymbrium officinale*	Hedge mustard	[86]
Primary dormancy	*Spergula arvensis*	Spurry	[87]
Primary dormancy	*Stylosanthes humilis*	Pencil flower	[88]
Primary dormancy	*Trifolium subterraneum*	Subterranean clover	[65]
Primary and secondary dormancies	*Xanthium pennsylvanicum*	Cocklebur	[66,67,68,69]

**Table 3:** Germination percentages obtained after 7 days at 15 °C with dormant (freshly harvested) and non-dormant (after 6 months of dry storage at 20 °C) sunflower embryos in the presence of water and in air (control), exogenous ethylene 50 μL L^−1^, ACC (1 mM), inhibitors of ethylene synthesis (AOA 1 mM; CoCl_2_ 5 mM), or ethylene action (STS 1 mM; 2,5-NBD 6.6 mL L^−1^). ACC, 1-aminocyclopropane-1-carboxylic acid; AOA, amino-oxyacetic acid; CoCl_2_, Cobalt chloride; STS, silver thiosulfate; 2,5-NBD, 2,5-norbornadiene. Means of four replicates ± SD. Values having the same letter are not significantly different at the 0.05 probability level, as determined by Duncan’s test. Modified from [57,58,89].

	Germination (%) in the Presence of						
	Air	C_2_H_4_	Precursor of C_2_H_4_	Inhibitor of C_2_H_4_ Synthesis		Inhibitor of C_2_H_4_ Action	
Embryos	Water	Water	ACC	AOA	CoCl_2_	STS	2,5-NBD
Dormant	18 ± 8 a	99 ± 1 b	98 ± 2 b	10 ± 5 a	15 ± 4 a	10 ± 4 a	0 c
Non-dormant	98 ± 1 b	100 b	100 b	50 ± 7 d	48 ± 5 d	78 ± 4 e	0 c

**Table 4:** Dormancy and ABA sensitivity of various mutants of *Arabidopsis thaliana* affected in the ethylene signaling pathway. ctr1, constitutive triple response 1; ein2, ein4, ein6, ethylene insensitive 2, 4, 6; etr1, ethylene response 1. Modified from [31].

Mutant or Transgenic Lines	Gene/Locus	Seed Dormancy	Hormone Sensitivity	References
*etr1-1*	*ETR1*	Enhanced	C_2_H_4_ insensitive	[59,60,61,178,179,181,186,187,188,189]
*etr1-2*	*ETR1*		ABA hypersensibility	
*etr1-3*	*ETR1*	Enhanced	Reduced C_2_H_4_ sensitivity	
*etr1-6*	*ETR1*	Slighly enhanced	More sensitive to ABA	
*etr1-8*	*ETR1*	Enhanced	-	
*ein2-1*, *ein2-5*, *ein2-49*	*EIN2*	Enhanced	ABA hypersensibility	[178,181,187,188,189]
*ein4-4*	*EIN4*	Enhanced	-	
*ein6*	*EIN6*	Enhanced	ABA hypersensibility	
*ctr1-1*, *ctr1-10*	*CTR1*	Early germination	Reduced ABA sensitivity	[178,181,186,188,189]

## Data Availability

This paper is a review and the reader can read all the citations in the reference list.
